# A Case of Aneurism of Thoracic Aorta

**Published:** 1884-05

**Authors:** A. M. Barker


### Clinical Reports.

### A Case of Aneurism of Thoracic Aorta.

Reported by A. M. Barker, M. D.

O. B., German, single, 50 years of age, and by occupation an artist, came under my care early in December, 1883, with the following history: For about one year he had been troubled with what he considered to be dyspepsia, and had been treated as a private patient, for that disease, at one of our public hospitals for several weeks. The first of November last, receiving no benefit from treatment, he left and took up his abode in a boarding house. He consulted several physicians and his disease was variously diagnosed as dyspepsia, Bright's disease, consumption and heart disease.

I found that he was vomiting his food, suffering severe burning sensations in his stomach; that at night he suffered very severe pain between the scapulæ, which disappeared during the day; that he had a severe cough, attended with expectoration of matter of a bluish-white color; that he had severe paroxysms of dyspœna. The bowels were constipated and the quantity of urine passed estimated to be normal. Some ten years ago he had a sore on his penis, which followed a suspicious connection, but healed within a week after its first appearance by the application of some simple caustic. No history of the secondary or tertiary symptoms of syphilis could be obtained. He had never had a rash about his body, his throat had not been sore, nor had his hair ever fallen out. He did not suffer from headache. The pulse was normal in frequency and character and there was no acceleration of temperature. Parents both died at an advanced age, and no history of any hereditary disease could be obtained. He was emaciated, face of sallow color and features pinched. The tongue presented a normal color. The respirations were a little difficult, but were not hurried. The inspiratory act seemed to be prolonged. An examination of his chest, by inspection, revealed in the infra-clavicular region—on the right side, about one inch from the sternum, between the first and second ribs—a violent pulsation, which could not, however, be called a pulsating tumor. This pulsation was more evident upon palpation, but was not attended by any peculiar whirring sensation. Posteriorly, no pulsation could be detected. Auscultation failed to give an aneurismal bruit, but a slight blowing sound was heard over the pulsating point mentioned. Posteriorly, no pulsation or sound could be detected. The respiratory murmur was normal, except on the left side, where it was heard less distinct than on the right. The heart sounds were normal. There was no œdema of the extremities and the abdomen was not distended. The area of dullness over the liver was normal, and no tenderness was found over that organ, nor the stomach. Some disease of the aorta was diagnosed, probably aneurism, and the patient placed upon potass. iod. gr. v, tr. belladonna gtt. x, and elixir calisaya ʒi, three times daily, after meals. He was also ordered to take, at bed-time, one grain of codeia, and to repeat every three hours until asleep or free from pain. To overcome the constipation he was given a teaspoonful of Carlsbad salts each morning, and a bread and milk diet, with beef tea made from Liebig's ext., and Hungarian wine ordered. The cause of his indigestion was well explained, as he was about to take his evening meal at the time of my visit. I noticed he had ham, fried potatoes, potato salad, a large slice of Bologna sausage and limburger cheese, and a cup of tea. He said he could not eat what was brought to him, and often sent it away untouched. The landlady, therefore, was carefully instructed in regard to his diet. A specimen of urine was requested to be sent to my office. An examination showed it free from albumen or sugar, and of normal specific gravity.

In two weeks' time he was improved so that he could attend to his business in his room, which was preparing pictures on stone for engravers. The first week in January he visited Niagara Falls in company with a friend and insisted that he walked ten miles, and felt first rate. As he was simply anxious to get well enough so that he could go to Germany to die, he was advised to take passage at once for home, but as he was better, and able to go to his work each day, he preferred to take his chances a little longer here. Early in February he called at my office and was suffering from severe dyspnœa. An examination revealed sonorous and sibilant rales over the whole of the posterior and part of the anterior portions of the chest. He was given an expectorant mixture in combination with Hoffman's anodyne, under which he improved and again resumed work. About the middle of February I was called by telephone to attend a patient who had started for my office but was taken sick on the way and could get no further. Upon my arrival, I found it was my patient with the aneurism. He felt well when he left his boarding house, but was attacked suddenly with what he said was a fainting spell, and obliged to lie down. After a short time he was revived sufficiently to get into my sleigh and was driven home. Brandy and aromatic spirits of ammonia were prescribed, and rest in bed. But as he felt so well he went to his place of business and resumed work.

He continued quite well till the 5th of March. On the evening of that day I was summoned in haste and told that he was dying. I found him in bed breathing with very great difficulty. The face, head, hands and upper part of the chest livid. The blood vessels were distended and protruded very prominently, particularly on the forehead. The pulse was full and hard, and beating about 120 times per minute. It seemed as if he could live but a very short time. Stimulants were administered and an anodyne given. The next morning he appeared comparatively comfortable; the breathing was much easier and it almost seemed that he had a new lease of life. He continued comfortable through the day, but at night another paroxysm came on, more severe, if possible, than the first. During my absence Dr. Hopkins was called, and the paroxysm was so severe that it was found necessary to administer chloroform, which gave relief so that he rested comparatively comfortable through the night. The next morning, early, I was summoned in haste and found him suffering from another paroxysm of dyspnœa, more violent, if possible, than the others. An anodyne was given in the form of a hypodermic injection of morphine, gr. ¼, which gave relief for several hours, when he was again taken with the dyspnœa and died suddenly.

Autopsy, twenty-four hours after death: Rigor mortis, well marked; brain not examined. The liver, stomach, kidneys and spleen were found to be healthy. There was a small quantity of urine in the bladder, estimated at three ounces. Examination of thoracic cavity: The right lung was found to be healthy; the left was congested, and the mucous membrane of the bronchia was red and contained a small quantity of mucus. There was no softening, nor evidence of inflammation, besides that seen in the bronchia. The heart was enlarged and the muscular tissue soft and friable. The valves were healthy. The aorta was immensely dilated, and at the commencement of the descending portion of the arch a large tumor was observed. This was found to be firmly adherent to the bodies of the first four dorsal vertebræ and corresponding ribs, which were eroded, and a portion of their tissue absorbed. These formed the posterior wall of the tumor, so that it was adherent closely to the bone. It therefore became necessary to tear it away in order to remove the aorta and heart. As this was done, a large clot escaped, the examination of which showed that it gradually obstructed the circulation through the aorta until it became almost complete and the patient died.

Several thoughts occur in connection with the case:

First—The correct diagnosis of the disease was overlooked by several practitioners, I am convinced, from the fact of a careless observation of symptoms and neglect of a thorough physical examination.

Second—This occurred in one of our large public hospitals.

Third—That death occurred from the effect of the tumor upon the circulation, and not upon its pressure upon neighboring organs, nor from the almost universal cause of death, viz.: spontaneous rupture.

Fourth—An effort was made by nature to cure the disease by solidification of the sac by means of coagula, the location of the clot and adhesion of the tumor to the thoracic walls verifying this conclusion.

